# Overcoming the barriers to women's leadership in eye health

**Published:** 2025-03-07

**Authors:** Sumrana Yasmin, Esmael Habtamu, Jennifer Gersbeck

**Affiliations:** 1 Deputy Technical Director – Eye Health: Sightsavers, Islamabad, Pakistan; 2 Chief Executive Director: Eyu-Ethiopia, Bahir Dar, Ethiopia; 3 Assistant Professor: International Centre for Eye Health, LSHTM, London, UK.; 4 Executive Director – Influence and Scaling Impact: The Fred Hollows Foundation, Melbourne, Australia.

**The shortage of female leaders in eye health is a matter of opportunity, not competence.**

**Figure:**
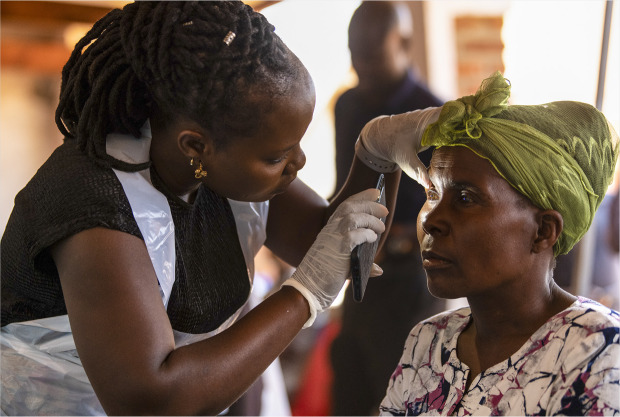
Deliberately create leadership opportunities for women working in eye health. malawi

© SIGHTSAVERS/THOKO CHIKONDI CC BY-NC-SA 4.0

What is holding women back from leadership positions in eye health is the lack of opportunity, not competence. There is evidence showing that, if women get the opportunity to lead, they excel in bringing change at global, national, and community levels.

However, there are many barriers that prohibit women from advancing to leadership positions, including:

- Systematic disadvantages, gender biases and stereotypes being perpetuated by society
- The inflexible nature of health system structures
- Economic disparities and inequitable access to professional development opportunities for women and girls, such as mentoring and skills development
- Discrimination, workplace bullying, and sexual harassment
- Lack of recognition and respect
- The gender pay gap – i.e., fewer women in high-paying jobs – which contributes to women becoming demotivated.

**Maggie Mukuka**

**Maggie Mukuka** is the Clinical Ophthalmic Nurse In charge of the eye department which she helped set up at Chilenje level 1 Hospital, Zambia in 2017. Maggie initiated the registration of ophthalmic nursing in Zambia, and provides ongoing mentorship to female eye health professionals in her field. She was recently recognised by the College of Ophthalmology of Central, Eastern, and Southern Africa (COECSA), speaking at the 2024 COECSA congress in Zimbabwe as a Women Leader Grant awardee.

“Having more women leaders in eye health reduces avoidable blindness and promotes gender equity,” according to Maggie. To make a difference, Maggie recommends building awareness campaigns on social media platforms regarding the importance of gender equity in eye health, and having quarterly engagement meetings to discuss the data. Asked if she could give advice to her younger self, she said: “Focus on your desired goal, identify a mentor that will assist in your goal achievement and focus on networking and collaborating.”

**Figure:**
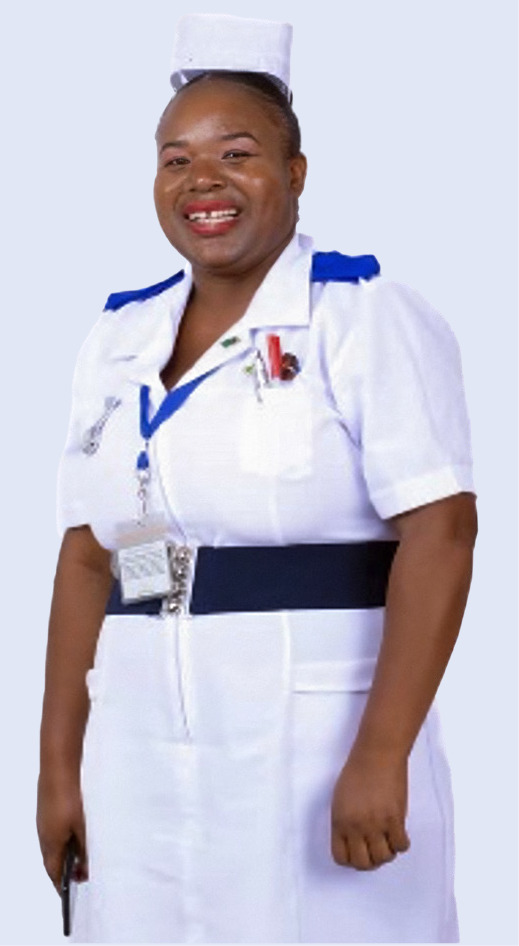

## Lessons from other sectors

Addressing these barriers requires long-term commitment, collaboration, effective and evidence-based strategies, and innovative and intentional interventions to bring a transformative change. A recent report^1^ identified the following lessons from other sectors on how to boost women's leadership in eye health.

### Deliberately create opportunities for leadership

Develop gender equity policies that encourage the involvement of women in leadership positions. For example, set up policies of equal representation of men and women on boards, as board chairs, on decision-making committees, and on recruitment panels.

### Monitor progress

Be clear about the change you are trying to make – i.e., set explicit targets. Then set up the right mechanisms to measure, monitor and communicate progress. The IAPB Gender Equity in Eye Health survey tracks a number of metrics, and might be a useful place to start (see the IAPB Gender Equity toolkit at bit.ly/IAPBgender).

### Provide support

Set up mentorship and sponsorship programmes specifically designed for aspiring women leaders, from an early career stage.

- **Mentorship** from a more senior colleague can provide women with career guidance, networking opportunities, and feedback and support to take on new roles and greater levels of responsibility.
- **Sponsorship** goes beyond the usual role of a mentor. Sponsors are senior colleagues who can use their position and influence to proactively advocate for women's advancement, for example, nominating them for positions in leadership groups. This will help women to advance their leadership career and be at the head of the table when the time is right.^2^

### Fix the problem, not women

Building the capacity of women as leaders is insufficient if systems aren't changed, as women themselves are not the limiting factor. Even women in leadership positions cannot change the system independently, as evidenced by research and case studies that consistently highlight the need to address institutional and structural barriers.^3^

Removing structural barriers that may hold women back from achieving their leadership potential could include progressive organisational policies like flexible working (e.g., having flexible start and end times to the working day, and/or working from home some of the time), and forums for women to share their lived experiences. Supporting women to balance their paid work and unpaid parenting or caring roles could involve offering shared parental leave, childcare support, and women's health benefits.

Note that women may face additional disadvantages related to age, disability, or ethnic group; additional supportive policies should be developed in collaboration with these groups.

**Wanjiku Mathenge**

**Wanjiku Mathenge** is the Co-founder and Director of the Rwanda International Institute of Ophthalmology and was recently appointed President of the African Ophthalmology Council.

In 2003, while working as an ophthalmologist in Nakuru Eye Clinic in Rwanda, Wanjiku noticed that her surgical lists always had men first, then women. Because of shortages of supplies, a lot of the time the surgeons didn't get to the women.

“I thought, ‘Why is it like this?’ What finally triggered me into action was a couple who came together; both were blind. The man, of course, was on the list – and we didn't get to the woman. The next day, the man was expecting his blind wife to still look after him. He didn't want to be discharged without his wife, even though the wife was still blind. He said, ‘Who's going to cook for me when I get home?’”  

“After that, I said that all men must bring their wives for a checkup before I do any surgery on the men. And my surgical list must always start with women.” This flipped the data for this district: the rapid assessment of avoidable blindness (RAAB) survey for Nakuru, conducted two years later, found lower point prevalence estimates of blindness and moderate to severe visual impairment in women than in men.

**Figure:**
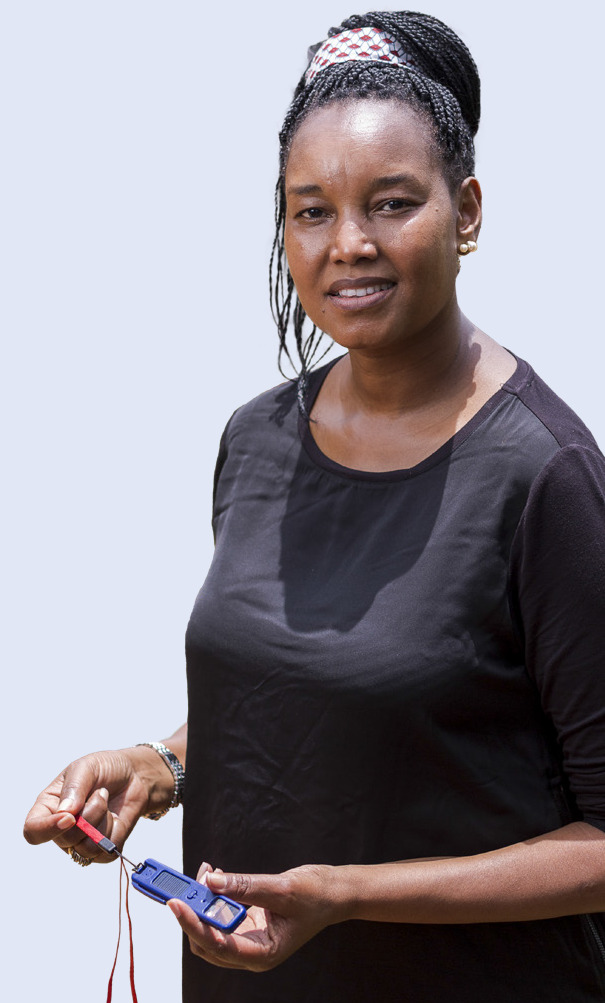

**Suma Ganesh**

**Suma Ganesh** is Director, Paediatric Ophthalmology at Dr Schroff's Charity Eye Hospital, New Delhi, India. She championed the creation of a flexible paediatric ophthalmology fellowship that allowed women to develop a specialty without compromising their family commitments.

As a result, the hospital has been able to recruit and train two full-time female paediatric ophthalmologists. “We need to be flexible with the training we offer,” she says. Dr Ganesh believes that women should support each other: “Behind every successful woman is another successful woman,” she says. “When an opportunity comes our way, we need to grab it and not let it go. We need to focus on our career growth and work together to remove any obstacles.“

**Figure:**
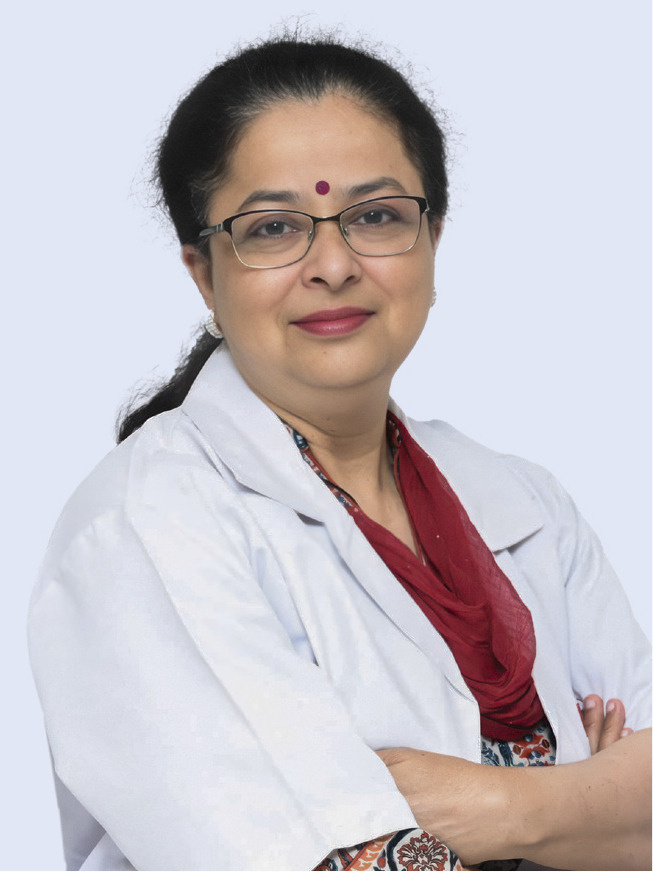

### Encourage men to support gender equity

Men in positions of power and influence should be fully involved as strong allies in the development of women and fill the development gap by offering opportunities, mentorship, and sponsorship.

Men can help to bring about cultural change by breaking and challenging systemic and cultural biases toward women and contributing to a gender-inclusive environment.

“Men can help to bring about cultural change by breaking and challenging the systemic and cultural biases toward women.”

Men can also help to break the ‘maternal wall’ of workplace discrimination against working mothers by eliminating the assumption that working mothers are less motivated to be involved in leadership positions than men,^3^ and advocating for a flexible and supportive working environment.^4^

Getting men in leadership positions onboard, however, requires strong advocacy and training on gender equity issues. A study showed that trained male executives were more likely to speak about gender inequity than their female counterparts.^5^

In conclusion, there are clear steps organisations and individuals can take to increase the proportion of women in eye health leadership positions. What we need most are people and organisations willing to take these steps.

## References

[1] The Fred Hollows Foundation. (2023). Women Deliver Eye Health; Let's Reframe Who Leads It.. http://bit.ly/WDEHfhf.

[2] Novotney A. (2023). Women Leaders MAKE Work Better.. Here's the Science Behind How to Promote Them..

[3] Roche. (2024). Women at the Helm of Healthcare Leadership: Why It Matters.. http://bit.ly/4hRFkWP.

[4] Lee H. (2023). 5 Ways to Improve the Representation of Women in Leadership Positions..

[5] Mattingly VP. (2018). Glass kickers: training men as allies to promote women in leadership: Colorado State University..

